# Supplementary material for: Voltage-gated proton channels from fungi highlight role of peripheral regions in channel activation
Source: Commun Biol. 2021 Feb 26;4:261. doi: 10.1038/s42003-021-01792-0 (PMC7910559; doi:10.1038/s42003-021-01792-0)
Supplement: Supplementary file 2 — Supplementary Information [file 42003_2021_1792_MOESM2_ESM.pdf]

Voltage-gated proton channels from fungi highlight role of peripheral regions in channel activation

Chang Zhao[1] and Francesco Tombola[1]

[1]Department of Physiology and Biophysics & Chao Family Comprehensive Cancer Center, University of California, Irvine, CA 92697, USA

SUPPLEMENTARY FIGURES 1-6

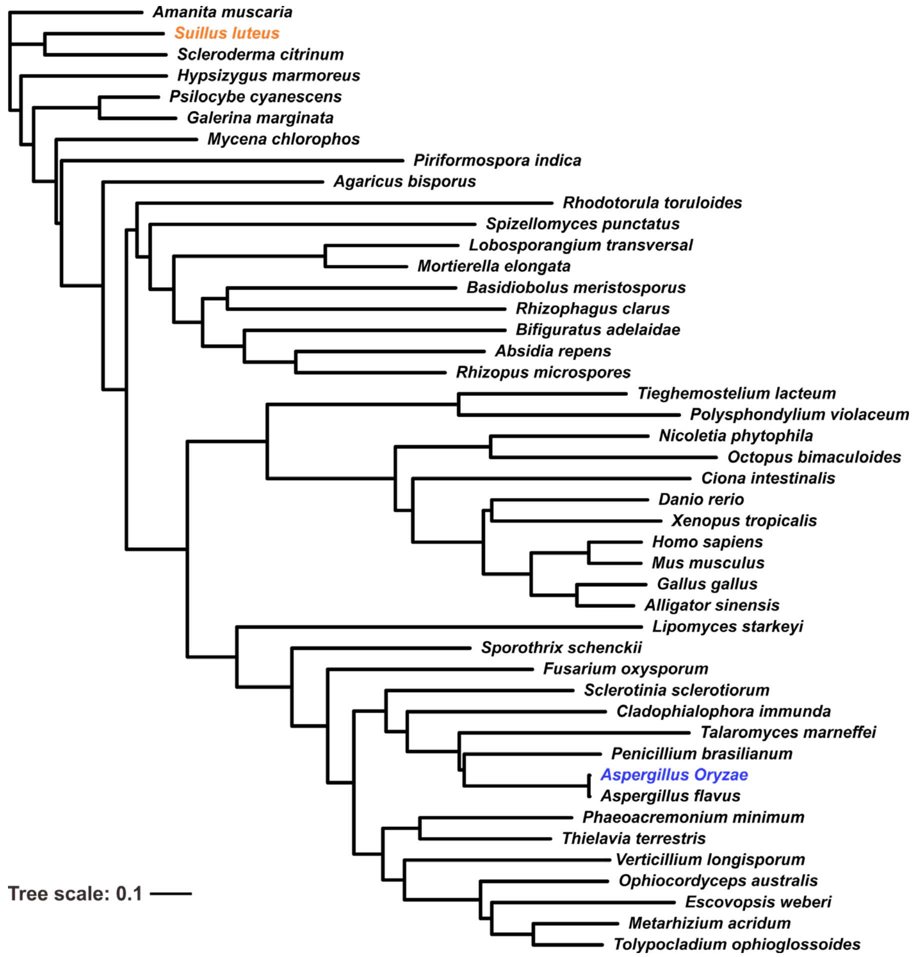

Supplementary Figure 1. Phylogenetic relationship between Hv1 channels from fungi and animals. Organisms from the kingdom Fungi includes representatives from *Ascomycota, Basidiomycota, Chytridiomycota, Zygomycota and Glomeromycota* divisions (see "Methods" section for sequence IDs and details). Tree scale 0.1 = 10 % difference between sequences. The same animal species from the cladogram of Fig. 1a were included in the phylogenetic tree. Representatives of slime molds (*Tieghemostelium/Dictyostelium* and *Polysphondylium* species) are also included here. These organisms used to be considered part of the Fungi kingdom, but they are now classified as protists.

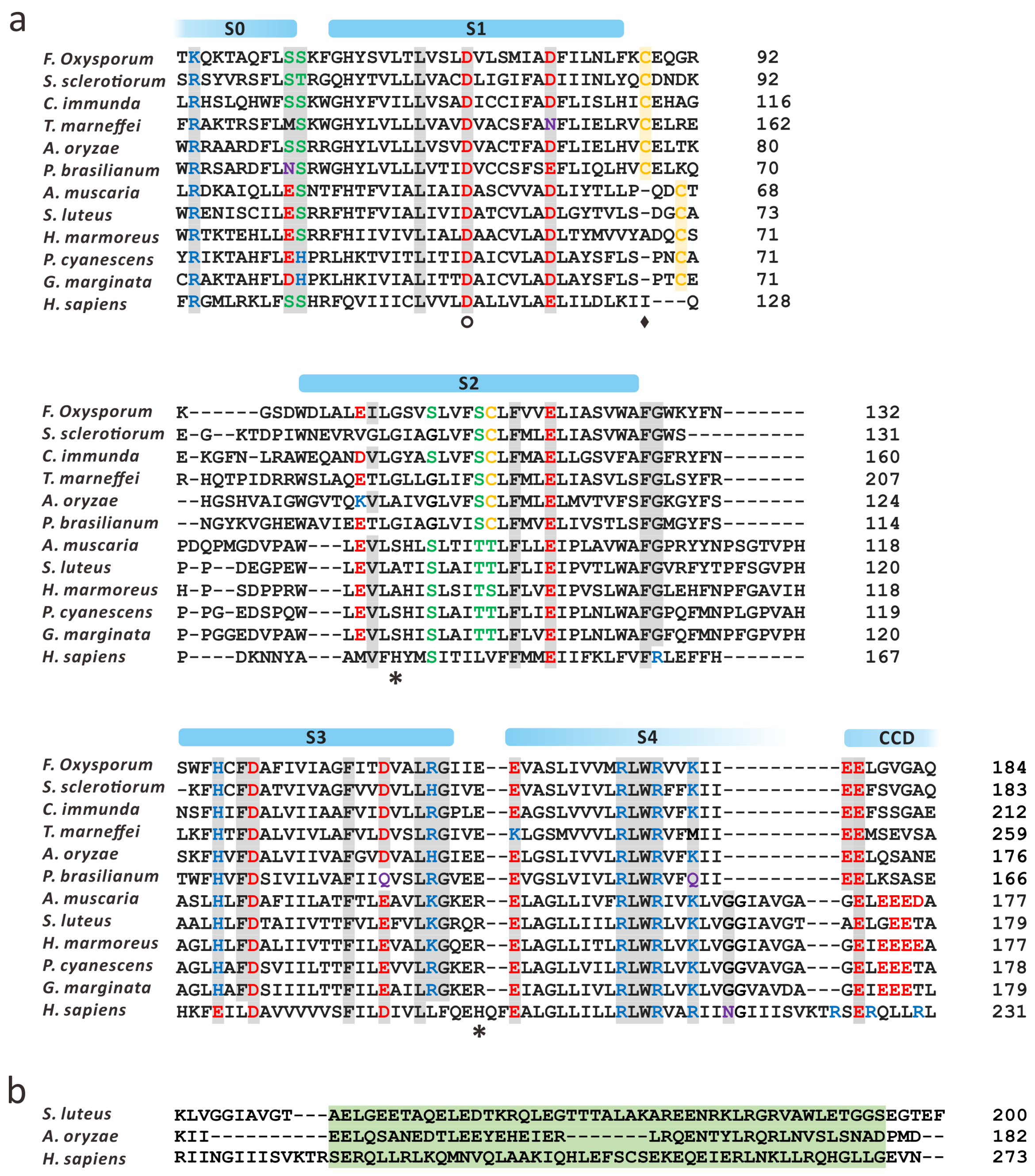

Supplementary Figure 2. Sequence alignment of proton channels from multiple fungal species. a) Comparison focused on VSDs of Hvs from *Ascomycetes* and *Basidiomycetes* in relation to human Hv1. °Aspartate residue known to be part of the selectivity filter of hHv1. *Histidine residues proposed to coordinate Zn^{2+} in hHv1. ◆I127: when a cysteine is introduced at this position in hHv1, it forms a spontaneous intersubunit disulfide bond. Endogenous cysteines in the S1-S2 loops of fungal Hvs are highlighted in yellow. b) Highlighted in green are regions predicted to form a CCD in SlHv1 and AoHv1 in relation to the CCD of hHv1 (estimated from 3VMX).

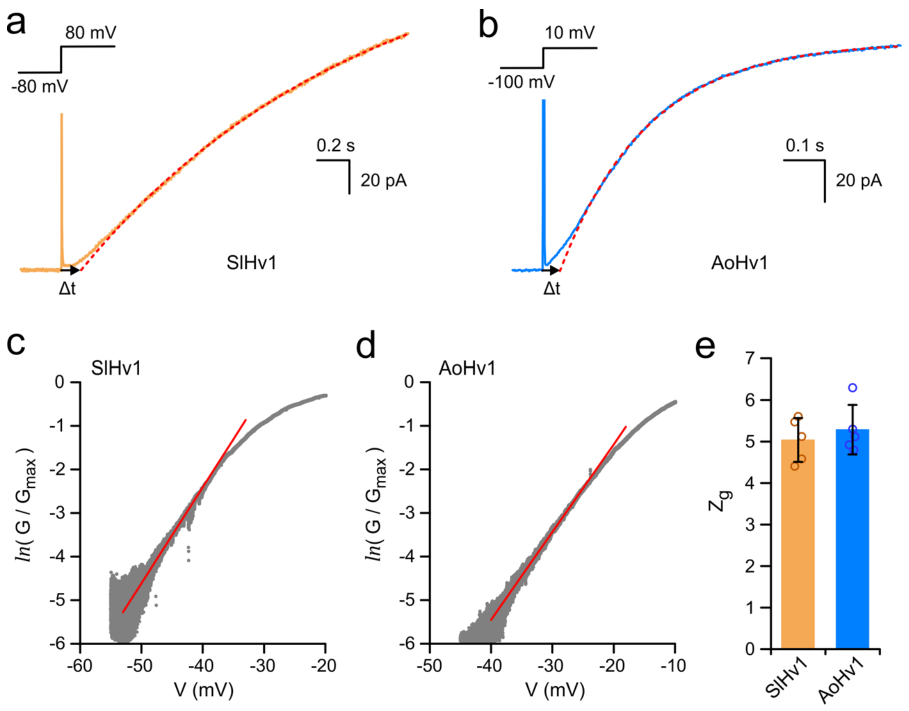

Supplementary Figure 3. Voltage-dependent opening of fungal Hvs. a-b) Examples of the initial phase of the time-course of activation for SlHv1 (a) and AoHv1 (b) in response to the indicated voltage steps ($pH_i = pH_o = 6.0$). Single exponential fits of the current traces, after the initial sigmoidal phase, are shown as red dotted lines. Black arrows indicate $\Delta t = t_e - t_o$, where t_e is the time at which the current extrapolated from the fit is zero, and t_o is the time of the transition in membrane potential. A $\Delta t > 0$ indicates that the channel spends time transitioning through closed states before opening. In these examples, Δt was 123 ms for SlHv1, and 40 ms for AoHv1. **c-d**) Representative plots for the determination of the gating charge (z_g) based on the limiting slope method. *ln*(G/G_{max}) was calculated as described in the "Methods" section. The linear fit, showed as red line, was performed between the ordinate range -5 to -4. Measurements were carried out in inside-out patch configuration, $pH_i = 5.5$, $pH_o = 6.5$ for SlHv1; $pH_i = pH_o = 6.0$ for AoHv1. **e**) Quantification of the gating charge (z_g) associated with voltage-dependent activation of the indicated channels. z_g values were derived from the slope of linear fits like those shown in (c) and (d), see "Methods". Each bar represents a mean value from five independent measurements. Error bars are SD.

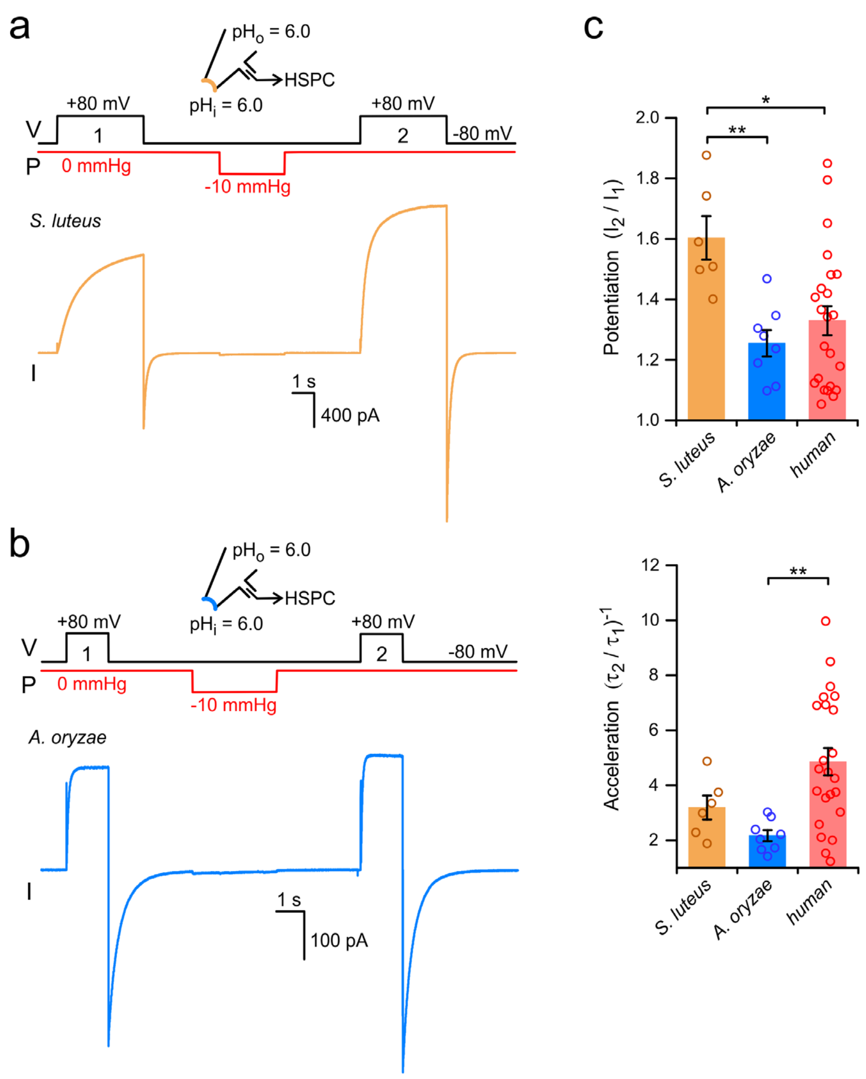

Supplementary Figure 4. Hv1s from *S. luteus* and *A. oryzae* are sensitive to mechanical stimulation. a-b) Examples of proton currents elicited by membrane depolarization for SlHv1 (a) and AoHv1 (b) before (step 1) and after mechanical stimulus (step 2). Change in membrane tension was induced via negative pressure applied to the patch pipette. The mechanical stimulus was delivered at resting membrane potential to inside-out patches. **c**) Averaged increases in current (potentiation) and activation rate (acceleration) caused by the mechanical stimulus (ΔP = -10 mmHg) in fungal Hv1s compared to human channel. Current values I_1 and I_2 were measured at the end of depolarization steps 1 and 2, respectively. Time constants from mono-exponential fits of current traces were used to calculate acceleration in channel activation $(\tau_2/\tau_1)^{-1}$. Each bar represents the mean of at least 6 independent measurements ± SEM. One-way ANOVA with Tukey's post-hoc tests were used for statistical analysis: *$p < 0.05$, **$p < 0.01$. Reference values for hHv1 are from Pathak et al. 2016 ([51]).

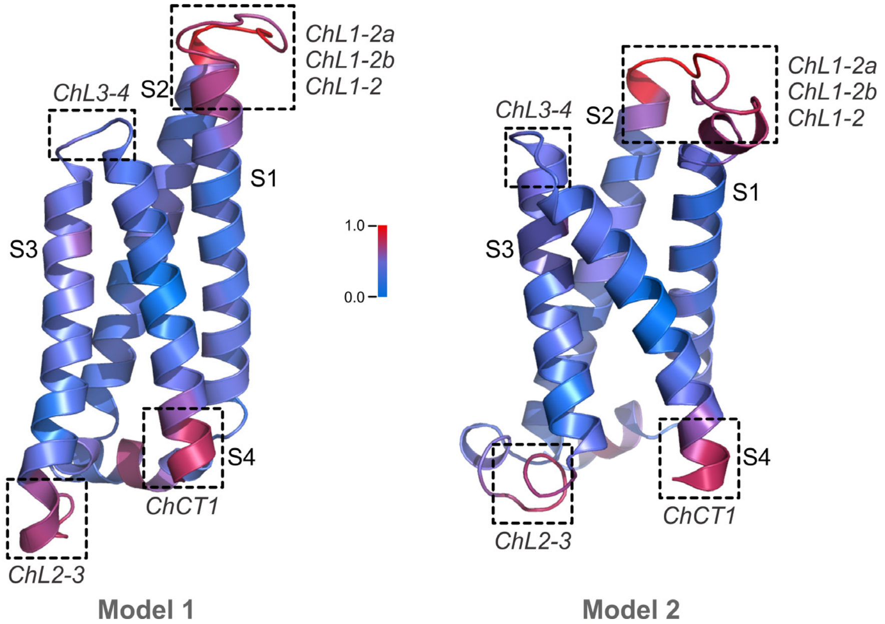

Supplementary Figure 5. Alternative structural models for the VSD of SlHv1. Divergence in sequence homology between SlHv1 and AoHv1 mapped on two alternative models of the SlHv1 VSD. Model 1 is based on the structure of mHv1cc (3WKV:A). Model 2 is based on the structure of CiVSP-VSD (4G80:I). Color gradient varies from minimal divergence (blue) to maximal divergence (red) (same as Fig. 5a). Dashed boxes indicate regions with the largest sequence divergence which were targeted by chimeragenesis. In both models these regions include: the S1-S2 loop and the outermost portions of helices S1 and S2, the part of the S2-S3 loop closer to helix S3, the S3-S4 loop, and the innermost portion of helix S4. Local differences between the two models can be seen in all the divergent regions; the most noticeable involves the transition between the S2-S3 loop and helix S3 (region targeted in the ChL2-3 chimera).

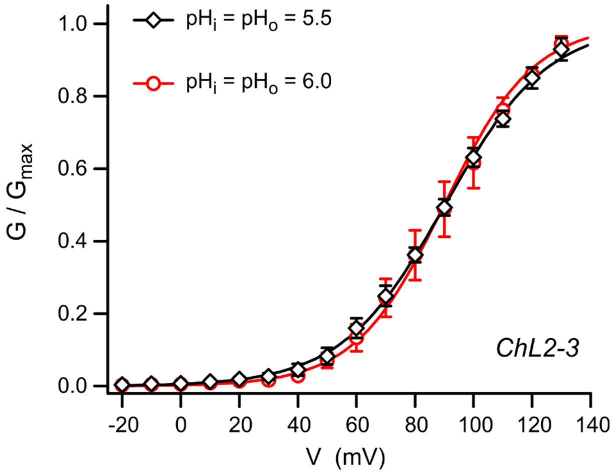

Supplementary Figure 6. Reduced pH sensitivity of the G-V relationship of chimera ChL2-3 under symmetrical conditions (ΔpH = 0). G-Vs under the indicated pH conditions represent the mean of 5 independent measurements. Error bars are SEM. The following G-V parameters were derived from Boltzmann fits of the data: $V_{1/2}$ = 90.2 ± 1.6 mV, slope = 17.7 ± 1.8 mV for $pH_i = pH_o$ = 5.5 (n = 5), and $V_{1/2}$ = 89.9 ± 3.9 mV, slope = 15.3 ± 1.7 mV for $pH_i = pH_o$ = 6.0 (n = 5). The negligible change in $V_{1/2}$ is to be compared to the corresponding change observed with SlHv1 WT ($\Delta V_{1/2}$ ~ 7.6 mV) from Fig. 3j.
