# Supplementary material for: Voltage-gated proton channels from fungi highlight role of peripheral regions in channel activation
Source: Commun Biol. 2021 Feb 26;4:261. doi: 10.1038/s42003-021-01792-0 (PMC7910559; doi:10.1038/s42003-021-01792-0)
Supplement: Supplementary file 3 — Description of Additional Supplementary Files [file 42003_2021_1792_MOESM3_ESM.pdf]

Description of Additional Supplementary Files

File name: Supplementary Data 1

Description: Dataset used to generate figures in the main text and supplementary figures.
